# Vitality Revisited: The Evolving Concept of Flourishing and Its Relevance to Personal and Public Health

**DOI:** 10.3390/ijerph20065065

**Published:** 2023-03-13

**Authors:** Alan C. Logan, Brian M. Berman, Susan L. Prescott

**Affiliations:** 1 Nova Institute for Health, Baltimore, MD 21231, USA; 2 Family and Community Medicine, University of Maryland, Baltimore, MD 21201, USA; 3 Medical School, University of Western Australia, Nedlands, WA 6009, Australia; 4 The ORIGINS Project, Telethon Kids Institute, Nedlands, WA 6009, Australia

**Keywords:** human flourishing, well-being, public health, high-level wellness, vitality, noncommunicable diseases, vitality, exposome

## Abstract

Human flourishing, the state of optimal functioning and well-being across all aspects of an individual’s life, has been a topic of philosophical and theological discussion for centuries. In the mid-20th century, social psychologists and health scientists began exploring the concept of flourishing in the context of health and high-level wellness. However, it is only in recent years, in part due to the USD 43 million Global Flourishing Study including 22 countries, that flourishing has entered the mainstream discourse. Here, we explore this history and the rapid acceleration of research into human flourishing, defined as “the relative attainment of a state in which all aspects of a person’s life are good” by the Harvard University’s Flourishing Program. We also explore the construct of “vitality”, which refers to a sense of aliveness, energy, and motivation; we contend that this has been neglected in the flourishing movement. We explore why incorporating measures of vitality, together with a broader biopsychosocial approach, considers all dimensions of the environment across time (the total exposome), which will greatly advance research, policies, and actions to achieve human flourishing.

## 1. Introduction

In the last five years, the concept of human flourishing has gained popularity and moved from academic circles of social and psychological sciences, where it was mainly studied as part of well-being and happiness research, into mainstream public discourse. Leading medical journals such as *The Lancet* have published opinion pieces underscoring the importance of “Human flourishing in a health-creating society” [1], and *The New York Times*, *The Guardian* legacy, and other legacy media outlets have published articles on the topic, with self-assessment quizzes and advice on ways to achieve flourishing [2,3,4]. In 2022, the Templeton World Charity Foundation [5], which has invested heavily in human flourishing research, hosted the first Global Scientific Conference on Human Flourishing [6]. The complexity and universality of flourishing is reflected in the diverse range of disciplines that have contemplated, examined, and debated its definition and determinants.

Here, in our narrative viewpoint, we consider the emerging science of flourishing in the context of the parallel emergence of “high-level wellness” and “exposome science”, and the wider biopsychosocial determinants of health. These historical aspects of flourishing are important as a means to highlight the struggle to define both health and flourishing, and the purpose of “medicine”, as either a decidedly narrow or broad discipline. It is our contention that the increasingly sophisticated understanding of flourishing requires the integration of social, psychological, and biological determinants, and is therefore, highly relevant to exposome science—the study of the total environment [7]. Similarly, while exposome science often has a dominant focus on *negative* or adverse exposures affecting health, this can be enhanced by placing greater emphasis on *positive* or buffering exposures that enhance health outcomes. Positive well-being has been shown to be a strong predictor of future health outcomes, even after accounting for depression or negative effects [8,9], and its *absence* is a predictor of depression [10,11]. Recent research suggests that positive mental health and mental illness are two distinct but interconnected domains of mental health, each with unique and common predictors that influence each other in complex ways [12].

In this context, levels of energy or “vitality” also appear to be a unique measure of mental and physical well-being [13]. Measurements of vitality, which emerged from the well-being literature, consider both negative (absence of fatigue) and positive (presence of energy) states [14]. There is evidence that vitality is a key determinant of health, happiness, positive relationships, and life satisfaction [15,16,17]. Yet, the measurement of vitality has so far been neglected in the emerging flourishing research. We therefore also contend that the flourishing movement would be greatly enhanced by the inclusion of “vitality” as a fundamental construct. We begin with a consideration of how concepts of human health have evolved in the last century.

## 2. From Health to Well-Being

“*Health is more than the absence of disease. It is something positive, and involves physique and vitality, and it is mental as well as physical. The inherent difficulty at the present time is the absence of scientific methods of measuring this positive element in health. Yet, the world knows as a matter of human experience that it is real and vital.*”

George C. Whipple, co-founder of Harvard School of Public Health, 1916 [18].

The promotion of vitality at both individual and community levels has been a long-standing goal for public and mental health experts. The 1948 World Health Organization (WHO) definition of health as “complete physical, mental, and social well-being” has influenced the study of human flourishing and its indicators. This emphasis on “well-being” set in motion a quest to better understand wellness and its biological, social, and psychological indicators. In 1952, a meeting of experts in medical education reached the following conclusion: *The goal of health now at mid-century calls for not only the cure or alleviation of disease. It calls for even more than the prevention of disease. Rather, it looks beyond, to strive for maximum physical, mental and social efficiency for the individual, for* [the] *family and for the community* [19]. The statement underscores that despite its importance, preventive medicine is not equivalent to a holistic, comprehensive, biopsychosocial approach that considers vitality and human potential.

Additionally, in the 1950s, public health physician and biostatistician, Halbert L. Dunn (1896–1975), promoted the idea of “high-level wellness” as part of the health continuum. This considered how individuals and communities maximize their fullest potential within the environment where they are functioning [20,21]. Dunn argued that even in the absence of disease, health is a complex state of overlapping levels of wellness and “*not a relatively flat, uninteresting area of ‘un-sickness’, but is rather a fascinating and ever-changing panorama of life itself, inviting exploration of its every dimension*” [22]. According to his model, the highest part of the dynamic wellness arc was “absolute vitality”, even if it was a short-term experience. He described this as a perception of harmony between the person and the external environment, accompanied by a feeling of being “*alive clear to the tips of your fingers. You have energy to burn. You tingle with vitality*” [23]. This perspective was aligned with that of Hans Selye, who first posited that various forms of stress siphoned off energy from the reservoir of available energy, a pool that, if depleted, would result in exhaustion and other states than health [24,25].

Dunn’s emphasis on wellness emerged in parallel with the increased understanding that individuals should be viewed as “bio-psycho-social beings” along the continuum of severe disease to peak wellness [26]. In 1957, the University of California psychiatrist Portia Bell Hume argued that whether individuals are considered ill or healthy, the approach to prevention, diagnosis and/or treatment should occur within “*a bio-psycho-social frame of reference*”, and that “*biological, psychological, social components in either mental health or mental illness cannot be disassociated with any attempt to understand and deal with so wide a range of illnesses and states of comparative health*” [27]. During this period, George L. Engel (1913–1999) further developed the biopsychosocial model of health [28]. While he critically analyzed the ways in which the dominant biomedical model determined the presence or absence of disease [28], his famed *Science* article, now cited over 18,000 times, was still largely “disease” focused. Engel was concerned about those who are “*told that they are in need of treatment when in fact they are feeling quite well*” and “*others feeling sick* [who] *are assured that they are well, that is, they have no disease*.” He argued that the *“biopsychosocial model which includes the patient as well as the illness would encompass both circumstances*” [29]. Despite the dominant focus on disease, Engel did point out that “health” does not lend itself to a simple definition, and he agreed that it is a dynamic state where an individual enjoys full social capacity [28].

Dunn, Engel, and the pioneering psychiatrist Hume all argued that the determinants of health include intersecting social, psychological, behavioral, and biological factors. The challenge was to learn more about the wide range of comparative health states. The development of Heimler’s social vitality questionnaire was an attempt to measure the global well-being of adults in the intersecting spheres of family, work, finance, friendship, and sexuality. This scale examined “satisfactions” and “frustrations” in these realms, and although the scale was reported to have clinical utility [30,31,32], its uptake by researchers was limited.

Since Dunn equated high-level wellness with the psychological construct of vitality, vitality scales became relevant to assessing comparative states of health (including subscales within validated questionaries, such as the Profile of Mood States and the SF-36). Vitality is measured by the level to which an individual is approaching life with excitement and energy; feeling vigorous and enthused; living life as an adventure; feeling alive and activated; and maintaining a zest for life. Recent studies show that vitality is a multifaceted, dynamic, and adaptive construct that can influence, and be influenced by, physical and psychological energy and stress [33,34,35,36]. Higher vitality scores are indicative of mental well-being, better physical health over the life-course, and a reduced risk of various non-communicable diseases [17,37,38,39,40,41,42,43]. Although the relationship between vitality and healthy lifestyle habits is complex and seemingly bidirectional [44,45,46,47,48], vitality predicts lower mortality independent to one’s lifestyle, depressive symptoms, baseline health status, and other sociodemographic factors [49]—as we will discuss further below.

Having a sense of purpose was also central to Dunn’s concept of high-level wellness [50]. Contemporary researchers have described purpose as the extent to which individuals experience life as being directed and motivated by valued life goals. Perceived purpose in life (PIL) is the extent to which life is motivated by personally valued goals and life aims. Purpose, along with comprehension (perceived coherence and understanding of their lives) and mattering (feeling their existence is of significance and value in the world), is considered to be a key component of meaning in life [51]. Much like vitality, meaning and purpose are linked to multiple positive health outcomes [52,53,54,55] and healthy lifestyle behaviors [56], independent of demographics, socioeconomic status, personality, prior history of disease, and lifestyle [57].

It was Dunn’s contention that a culture based on consumerism and materialism (such as his own in mid-century Americana) is at odds with the growth of empathy and altruism that otherwise encourage high-level wellness [50]. Since then, materialism has been linked with both depression and diminished psychological well-being [58,59], and these associations are cross-cultural [60]. Consistent with Dunn’s hypotheses, meaning in life appears to be an important protective factor in the relationship between materialism and depression [61,62].

## 3. From Well-Being to Flourishing

“*Do we really know what human good or human flourishing consists of, or do we…really have anything like an adequate conception of the social conditions for its attainment, or, if in turn we know that, do we know how to bring those social conditions into being?*”

Kai Nielsen in *Human Needs and Politics*, 1977 [63].

Academic considerations of human flourishing have a long history in philosophy, religious studies, and social psychology [64,65] (Box 1). The field of social work was one of the first to attempt to measure human flourishing in the 1960s, aided in part by the psychiatric social worker Eugene Heimler [66] and the development of a social vitality scale [67]. Heimer’s questionnaire aimed to evaluate the interaction of various life factors such as family, work, finance, and interpersonal relationships in terms of determining human flourishing, both for individuals and society—via “*the individual’s adaptation to* [their] *society, but also a society’s adaptation to individuals inside and out it*” [64].

Box 1 **Flourishing—Historical Definitions.**

The term “flourishing” comes from the Latin word “floreo” meaning to bloom. Historically, the words “bloom” and “flourish” were used interchangeably to describe a state of health, growth, success, and abundance. For example, in the nineteenth century, as “state of health and growth, promising perfection, a flourishing condition; a state full of life and vigor; a period of high success” and “[growth] in honor, comfort and happiness, or whatever is desirable; to be increased with good things or qualities; to grow and augment; to thrive” [68,69]. More recently, flourishing has been defined as “the relative of attainment of a state in which all aspects of a person’s life are good including the contexts in which that person lives,” by Harvard University’s Flourishing Program.

In the late 20th century, numerous health assessment scales were developed to track well-being [70,71]. However, these were fragmented and they tended to assess psychological and social well-being separately. A landmark 2002 study by Corey L.M. Keyes used both psychological and social well-being scales to examine the mental health continuum in an adult population of 3032 individuals. People who scored in the upper tertiles on emotional and social well-being scales (40 items) were considered to be “flourishing in life”, whereas those in the lower tertiles were considered to be “languishing”. The results showed that only 17.2% of this sample of US adults (without depression) were considered to be flourishing, while 12.1% were considered to be languishing [72]. It remains unclear whether those who do not meet the criteria for mental disorders but are considered “subthreshold” or “subsyndromal” are considered healthy but languishing [73,74,75].

Keyes proposed that the characteristics of flourishing can be identified as clusters of symptoms that represent an underlying state, similar to how symptoms of mental disorders cluster in diagnostic manuals [76]. His widely cited 2002 paper was a highly influential catalyst for flourishing research, bringing concepts of “flourishing” and “languishing” into the mainstream lexicon. Following Keyes’ publication, Barbara Fredrickson and Marcial Losada used his combined scales in a study of university students and found that flourishing was characterized by a consistent balance of positive emotions (such as joy, love, gratitude, awe, hope, etc.) over negative emotions (such as anger, fear, guilt, etc.) over time [77]. Other studies support Keyes finding of a low frequency of flourishing (14–21%) in young adults who are otherwise considered healthy [78,79].

In 2010, Diener and colleagues developed a more specific eight-item Flourishing Scale to assess psychological well-being through measures of self-perceived success in relationships, self-esteem, purpose/meaning, and optimism. One of the eight questions on the scale harkens back to traditional philosophical and theological underpinnings of human flourishing—“*I am a good person and live a good life*” [80]. It is noteworthy that the majority of citations referencing the Diener 2010 Flourishing Scale (on Google Scholar) have occurred since 2020, indicating a recent surge in scientific interest in the concept of human flourishing.

## 4. Consideration of Human Virtues, Character Strengths, and Social Resources

The assessment of flourishing is part of a growing list of validated well-being measurements, but many of these scales lack a clear theoretical foundation and have widely differing definitions of well-being, encompassing mental, physical, and social well-being [81]. In 2017, VanderWeele argued that the existing measures of flourishing had omitted direct assessments of health, financial stability, virtue, and the character strengths that are believed to be foundational to flourishing from philosophical and theological perspectives [82]. These concerns led to the development of the 12-item Secure Flourish questionnaire covering six domains—happiness and life satisfaction; health, both mental and physical; meaning and purpose; character and virtue; close social relationships; and financial/material stability.

VanderWeele’s argument that virtue is a crucial component of flourishing has been supported by an increasing number of studies linking character strengths to subjective well-being, healthy social relationships, success in academic and occupational settings, and healthy lifestyle behaviors [83,84,85]. Briefly, character strengths are positively valued personality traits that, while generally stable over time and context, can be influenced by specific cultural values, instruction, and interventions [86,87]. Among two dozen character strengths, honesty, fairness, kindness, judgment, and curiosity are highly endorsed in cross-cultural samples [88]. Character strengths are an avenue to six virtues that are valued across cultures: wisdom, courage, humanity, justice, temperance, and transcendence, which have been linked to well-being [89,90,91].

The exact ways that character strengths contribute to flourishing are not yet understood, but it is believed that individuals who exhibit these strengths develop strong social connections, experience emotional benefits from doing good, and make life choices that increase their chances of reaching their full potential. VanderWeele proposes that character strengths are both constitutive of flourishing and causal for factors associated with well-being [92]. Indeed, longitudinal research shows that placing a high value on character strengths predicts subsequent well-being [93]. In a recent longitudinal study, an orientation to promote good (consistent thoughts and taking actions that contribute to the good of oneself and others) was positively associated with multiple measures of well-being—including the subsequent life satisfaction and happiness, self-assessed mental health and physical health, social connectedness, and purpose, and meaning in life, as well as a lower risk of anxiety, loneliness, and depression [94]. These results are supported by separate longitudinal and multinational cross-sectional studies linking character strengths with health and overall quality of life [95,96].

Although biopsychosocial approaches to understand the health effects of the “total” environment (i.e., the “exposome”) are often championed, in practice, the “bio-psycho” dimensions typically dominate while the “social” determinants are neglected [97,98]. VanderWeele has contended that financial/material stability and security (food, housing, and safety) should also be considered as critical to the sustainability of flourishing over time [82]. The Secure Flourish instrument therefore measures financial stability and safety concerns by asking two questions—“How often do you worry about being able to meet normal monthly living expenses?” and “How often do you worry about safety, food, or housing?” [82,99]. This recognizes that financial stability, quality housing, nutritious food, clean water, and levels of individual/community safety are at once basic and complex interrelated determinants along the health continuum [100,101,102]. With this more comprehensive and nuanced understanding of the wider factors that promote or detract from human flourishing, the Secure Flourish index is an important advancement, underscoring the need for multi-disciplinary approaches for research and intervention (Box 2) [103].

Box 2 **Secure Flourishing Index [82].**

Domain 1: Happiness and Life Satisfaction.

1. Overall, how satisfied are you with life as a whole these days?
2. In general, how happy or unhappy do you usually feel?

Domain 2: Mental and Physical Health.

3. In general, how would you rate your physical health?
4. How would you rate your overall mental health?

Domain 3: Meaning and Purpose.

5. Overall, to what extent do you feel the things you do in your life are worthwhile?
6. I understand my purpose in life.

Domain 4: Character and Virtue.

7. I always act to promote good in all circumstances, even in difficult and challenging situations.
8. I am always able to give up some happiness now for greater happiness later.

Domain 5: Close Social Relationships.

9. I am content with my friendships and relationships.
10. My relationships are as satisfying as I would want them to be.

Domain 6: Financial and Material Stability.

11. How often do you worry about being able to meet normal monthly living expenses?
12. How often do you worry about safety, food, or housing?

## 5. Vitality as a Core Construct in Health and Well-Being

Levels of energy or “vitality” may be unique measures of mental and physical well-being that may not be captured through other questions about self-described health and well-being—including the current flourishing scales. Specifically, people who may score highly on measures of physical and social well-being may nonetheless report low levels of energy, and conversely, those who experience physical and emotional challenges may have high energy and vitality. Indeed, in one of the largest studies on vitality to date (n = 10,000), researchers reported that many of the individuals that they initially expected to have high vitality levels (based on youth and lack of health problems) actually experienced low vitality [104]. The authors concluded that this illustrates “*that the capacity to pursue life with health, strength and energy is multi-dimensional and personal*” [104]. It is our contention, based on the evidence described below, that vitality is a core component of what it means to flourish in life.

Vitality is a central consideration of the self-determination theory, a framework for understanding human motivations and personality, with emphasis on inherent growth tendencies and innate psychological needs [105]. The primary psychological needs are competence, relatedness, and autonomy, and the extent to which an individual is satisfied or frustrated in meeting those needs, vitality will be enhanced or suppressed. The self-determination theory contends that subjective vitality is not a simple reflection of arousal—rather, it represents the experience of energy available to the self. Put simply, an individual could be subjectively nourished and physically rested, yet lacking in spark and vitality [106].

The omission of vitality as a core construct of flourishing may potentially weakens the value of flourishing assessments. Neither of the current flourishing indexes (the 12- and 40-item versions of VanderWeele’s scale [107] or Diener’s Flourishing Scale) have included a specific assessment of vitality or a direct question concerning energy levels. As we outline further below, this is an important consideration for future effects to advance our assessment and understanding of flourishing.

In 2013, Huppert and So proposed that assessing vitality could help to round out Diener’s measures of flourishing and provide a more comprehensive picture of an individual’s subjective well-being—capturing vitality in the item “I had a lot of energy” over the previous week [13]. In their study of 43,000 people from 23 European countries, they found that measures of flourishing that included vitality were only modestly correlated with life satisfaction (e.g., “how satisfied are you with your life nowadays?”)—only one-third of those who reported high life-satisfaction were actually flourishing [13]. In a study of Egyptian adults (n = 423), subjective vitality was found to be an important mediator in the relationship between flourishing and life satisfaction [15]. This is also consistent with a separate study of Turkish university students, which identifies subjective vitality as a mediator between life satisfaction and happiness [16]. Vitality has also been found to be a mediator in the relationship between healthy lifestyle behaviors and healthcare and productivity (absenteeism) costs [108]. Among primary care patients, vitality has been found to fully mediate the relationship between hope and physical health, social relationships, perceived safety, and healthfulness of the local physical environment [17].

## 6. The Case for Including Vitality: More Comprehensive Assessment of Flourishing

The advantage of including vitality (as a form of self-reported energy) within flourishing assessments is that it reflects both physical and psychological wellness. Vitality is a unidimensional construct that considers both negative and positive states; that is, the presence of energy (energy as a positive asset) and the absence of fatigue (fatigue as a negative indicator/hinderance to vitality) [14]. Vitality also links integrated biopsychosocial theories of human functioning along a health continuum [109,110,111,112]. The research shows that vitality is positively associated with an individual’s quantity and quality of social contacts [113], and while vitality appears to be a factor in the promotion/maintenance of healthy lifestyle behaviors, intervention studies suggest that vitality is also a product of healthy dietary practices and regular physical activity [114,115].

In addition to physical and mental energy, vitality has been described in terms of “motivation” and “resilience” by health sciences—the desire to move toward goals and the capacity to respond to challenges, respectively [116,117]. In keeping with the Keyes concept of flourishing, as reflected in positive health “symptoms’ (the opposite of those associated with ill-health), the presence of energy (vitality) is the opposite of fatigue and tiredness. This is important because significant numbers (31–38%) of otherwise healthy populations report fatigue [115,118,119]. The absence of vitality and the presence of fatigue is a predictor of later non-communicable disease and mortality in prospective studies [120,121,122]. Yet, although fatigue is mentioned in about 20% of patient encounters in general practice, it is often ignored without follow-up [123,124].

In their study of 150 languages, VanderWeele and colleagues point out that vitality could be interpreted in many different ways according to human experiences [125]. They concluded that terms related to vitality settle around three main themes—spirit (inclusive of “life force”, “channels”, “soul”, and “transcendence”), energy (inclusive of “fortitude”, “channeling”, “willpower”, and “recharging”), and heart (inclusive of “desire”, “affection”, “passion”, and “satisfaction”) [125]. This is an important consideration, especially when determining the relationships between vitality as a broad construct, spiritual well-being, and the outcomes along the health continuum [126,127]. However, when viewed along the tighter lines of energy versus fatigue, vitality is among the least problematic aspects of the frequently used Short-Form Health Survey (SF-36), which is currently translated and in use in dozens of countries. This is likely because people across cultures understand, with careful translation, what it means to be tired and fatigued or to have energy [128,129,130,131,132].

It is not our suggestion that vitality is an equivalency of human flourishing. Rather, we propose its inclusion. For example, consider a socioeconomically disadvantaged person living in a marginalized community holding down two minimum wage jobs—that person could easily rate their physical and mental health as good, but if asked, could rate their energy levels as low and tiredness high. Is that person flourishing? Longitudinal research by VanderWeele and colleagues shows that meaning and purpose, social connectedness, financial security, and the single character item “*I always act to promote good in all circumstances, even in difficult and challenging situations*”, are most robustly connected to subsequent flourishing [107]. Given the background research on vitality, this indicates to us that a blending of energy assessments (or other aspects of vitality connected to ‘life force’) with virtue, meaning, and resource availability, along with basic questions about physical and mental health, will provide a more accurate approximation of composite flourishing.

## 7. Towards Greater Integration: From Biopsychosocial Research to The Exposome

There is a growing call for an integrated approach to health on all scales. This recognizes and addresses the interconnected factors that promote resilience and decrease vulnerability from the first moments of life—including the attitudes and value systems that govern these—for mutual benefits to individuals, communities, and natural systems upon which we depend [97,133].

The study of the total accumulated environmental exposures (both detrimental and beneficial) that can help predict the biological responses of the “total organism to the total environment” over time is referred to as “exposome” science [7]. Exposome science has been amplified by the new era of “omics” technologies—the ability to simultaneously measure large numbers of biomolecules representing functional proteins (proteomics), metabolites (metabolomics), gene expression (epigenomics and transcriptomics), and genetic influences [134]. These markers can illuminate the biological implications of the total lived experience of individuals and entire populations [135,136]. When combined at larger scales, these measurements can be analyzed with the aid of bioinformatics and biostatistics to predict personalized biological responses—while potentially enhancing large-scale health care in profound ways [137].

To advance flourishing research, especially to increase its relevance for clinicians and communities (through policy), an “exposome” perspective is required. When social psychologist Dr. Marie Jahoda wrote the first comprehensive review on positive mental health (as opposed to mental illness) in 1958, one prevailing criticism was a lack of the “biologic and physiologic components of mental health” [65]. Notwithstanding the dominance of biological aspects of disease in clinical settings, research on human flourishing still suffers from a dearth of information on the genetics and biology of wellness, human temperament and, by extension, character [138]. Put simply, exposome science can help illuminate the biological implications of the total lived experience of individuals and entire populations [7,139].

One major portion of exposome science concerns the microbiome—microbes and their functional genetic materials that act to “transduce” the total environment and the context, which is so important to the definition of flourishing [97]. Intervention studies using non-pathogenic microbes show that they can influence fatigue and human vitality [140,141,142], while studies show that the disturbance of the microbiomes (i.e., dysbiosis) is associated with a higher risk of both physical and mental diseases, disorders, and syndromes [143,144]. Both human and animal research shows that personality features and temperament can both influence, and be influenced by, the gut microbiome [145].

Taken as a whole, the emerging microbiome science challenges the idea of how the “self” is defined [146,147], or at the very least, since the physiological functions of humans and microbiomes are partly integrated, what it means to be human [148]. Socioeconomic disadvantage and marginalization, and the power structures that maintain inequity, are manifest in the human microbiome, a process known as “dysbiotic drift” [149,150]. The millions of dollars that are invested in human flourishing represent an opportunity to further understand the links between the upstream drivers of dysbiosis, the biological markers that illuminate structural inequalities, and unrealized human potential [97,151].

## 8. Future Directions

The long list of personality and behavioral factors mediating the associations between flourishing and health-related outcomes—ranging from dispositional awe to parenting styles—will undoubtedly grow [152,153]. At this point, a better understanding of the biopsychosocial factors that facilitate flourishing across cultures is imperative, including how to actively promote these.

Until recently, the research focus on subjective well-being and positive mental health has been on individuals and communities from affluent westernized countries. Although researchers are turning their attention to flourishing in the Global South, low- and middle-income countries, and culturally diverse populations, major gaps still remain [154,155,156]. Even in affluent societies, flourishing is not an equally distributed asset, which raises many urgent research questions about the structural impediments to flourishing [157,158]. It is also likely that diminished flourishing and increased languishing is contributing to the recent declines in life expectancy in the United States (and to a lesser extent, other countries), especially in socioeconomically marginalized groups [159,160,161]. Despite the identification of many biopsychosocial determinants of disease and life expectancy, these problems endure in the absence of policy interventions [162].

VanderWeele has underscored that ‘flourishing is a property of human beings and the contexts in which they are situated’ and should be ‘understood as the relative of attainment of a state in which all aspects of a person’s life are good including the contexts in which that person lives’ [163]. It is implicit within this definition that individual flourishing is dependent upon mutualism, compassion, justice, and empathy at wider group-level and institutional scales. Through this lens, VanderWeele contends that research into flourishing can give greater emphasis to the role of “character” in improving the “context”—which is to say the environment and all of its structures of power—to promote the good of all [163]. Although the current definitions of flourishing might not be precise, especially in what might be considered “good”, VanderWeele proposes that this ambiguity is an asset that allows for wider understanding of cross-cultural traditions and the embrace of what is valuable to humanity at large [163]. We agree with this assessment, and also wonder if future research might examine the extent to which “feeling alive”, or “energetic” would enhance a global definition of flourishing.

## 9. Conclusions

Academic interest in human flourishing has been longstanding within philosophy and theology. In the midst of declining longevity [164,165] and a growing mental health crisis [166,167,168], international heath–science researchers are now approaching the subject of flourishing with intense scrutiny. Aided in part by the USD 43 million Global Flourishing Study that included 22 countries and attracted media attention, the concept of flourishing—defined by the study group as *“the relative attainment of a state in which all aspects of a person’s life are good including the contexts in which that person lives”* [163]—has entered into the mainstream discourse, which is of high relevance to biopsychosocial medicine. Here, we have argued that the emerging flourishing movement has overlooked vitality as a fundamental construct. We also make the case that future research would be enhanced by examining flourishing through the lens of the exposome—the total lived experience—and that employing additional biological measures to identify pathways of influence may provide more integrated perspectives. Professionals working under the principles ideally positioned to help shape the direction of flourishing research, and by extension, the clinical relevancy and potential policy implications of this rapidly evolving effort.

## Data Availability

Not applicable.
